# Crystal structure of 2-chloro-*N*-(3-fluoro­phen­yl)acetamide

**DOI:** 10.1107/S2056989015007240

**Published:** 2015-04-18

**Authors:** S. Sreenivasa, P. A. Suchetan, S. Naveen, N. K. Lokanath, K. S. Srivishnu

**Affiliations:** a Department of Studies and Research in Chemistry, Tumkur University, Tumkur, India; b Department of Chemistry, University College of Science, Tumkur University, Tumkur 572 013, India; c Institution of Excellence, University of Mysore, Mysuru-6, India; d Department of Physics, University of Mysore, Mysuru-6, India; e University College of Science, Tumkur, India

**Keywords:** crystal structure, disordered F atom, *N*-aryl­amides, hydrogen bonding

## Abstract

In the title compound, C_8_H_7_ClFNO, the F atom is disordred over the *meta* positions of the benzene ring in a 0.574 (4):0.426 (4) ratio and the Cl atom is *syn* to the O atom [O—C—C—Cl = 5.6 (3)°]. A short intra­molecular C—H⋯O contact occurs. In the crystal, mol­ecules are linked into amide *C*(4) chains propagating in [101] by N—H⋯O hydrogen bonds.

## Related literature   

For compounds in which the *meta* fluorine substituent of a benzene ring exhibits positional disorder, see: Nayak *et al.* (2012 ▸); Sanjeevarayappa *et al.* (2015 ▸).
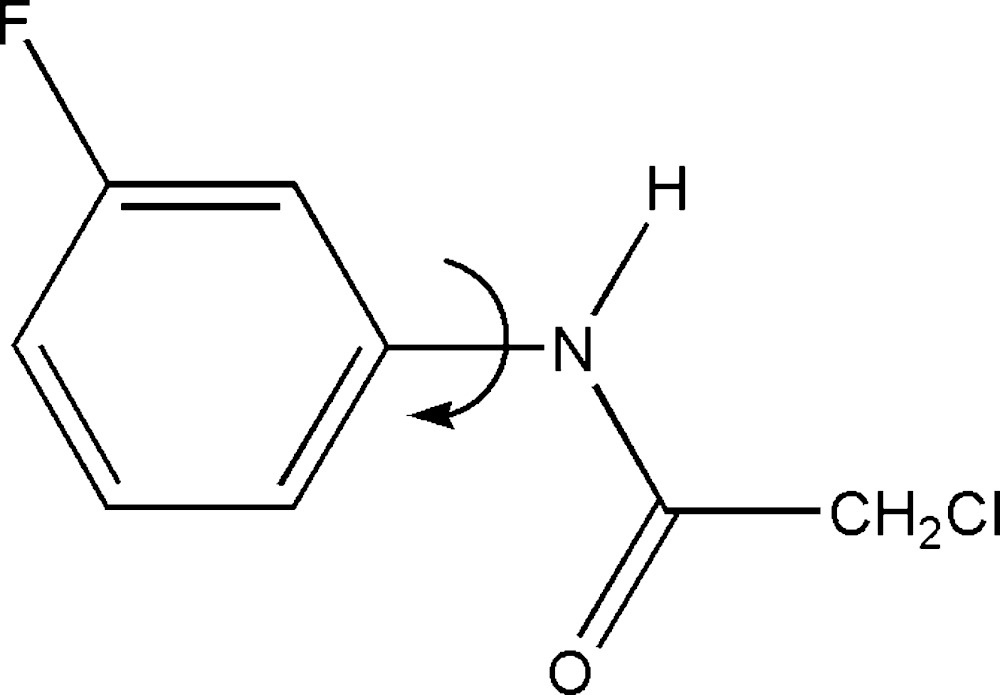


## Experimental   

### Crystal data  

C_8_H_7_ClFNO
*M* *_r_* = 187.60
Monoclinic, 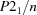

*a* = 5.0441 (2) Å
*b* = 18.2374 (7) Å
*c* = 8.8653 (3) Å
β = 99.843 (1)°
*V* = 803.53 (5) Å^3^
*Z* = 4
Cu *K*α radiation
μ = 3.95 mm^−1^
*T* = 293 K
0.31 × 0.24 × 0.19 mm

### Data collection  

Bruker APEXII diffractometer
Absorption correction: multi-scan (*SADABS*; Bruker, 2009 ▸) *T*_min_ = 0.368, *T*_max_ = 0.472
6045 measured reflections
1304 independent reflections
1297 reflections with *I* > 2σ(*I*)
*R*_int_ = 0.041
1 standard reflections every 1 reflections intensity decay: 1%

### Refinement  

*R*[*F* ^2^ > 2σ(*F* ^2^)] = 0.039
*wR*(*F* ^2^) = 0.105
*S* = 1.15
1304 reflections
123 parameters
1 restraint
H atoms treated by a mixture of independent and constrained refinement
Δρ_max_ = 0.26 e Å^−3^
Δρ_min_ = −0.25 e Å^−3^

### 

Data collection: *APEX2* (Bruker, 2009 ▸); cell refinement: *SAINT-Plus* (Bruker, 2009 ▸); data reduction: *SAINT-Plus*; program(s) used to solve structure: *SHELXS97* (Sheldrick, 2008 ▸); program(s) used to refine structure: *SHELXL97* (Sheldrick, 2008 ▸); molecular graphics: *Mercury* (Macrae *et al.*, 2008 ▸); software used to prepare material for publication: *SHELXL97*.

## Supplementary Material

Crystal structure: contains datablock(s) I. DOI: 10.1107/S2056989015007240/hb7400sup1.cif

Structure factors: contains datablock(s) I. DOI: 10.1107/S2056989015007240/hb7400Isup2.hkl

Supporting information file. DOI: 10.1107/S2056989015007240/hb7400Isup3.cml

**Fig. 1.**
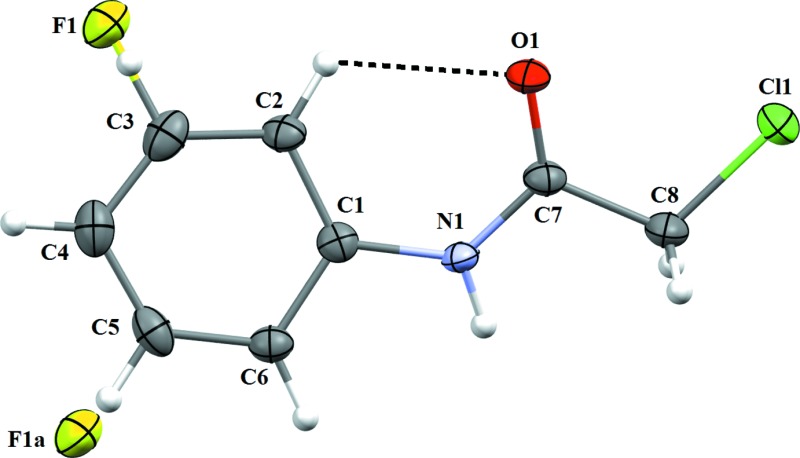
A view of the molecular structure of (I), with displacement ellipsoids drawn at the 50% probability level.

. DOI: 10.1107/S2056989015007240/hb7400fig1.tif
A view of the mol­ecular structure of (I), with displacement ellipsoids drawn at the 50% probability level.

**Fig. 2.**
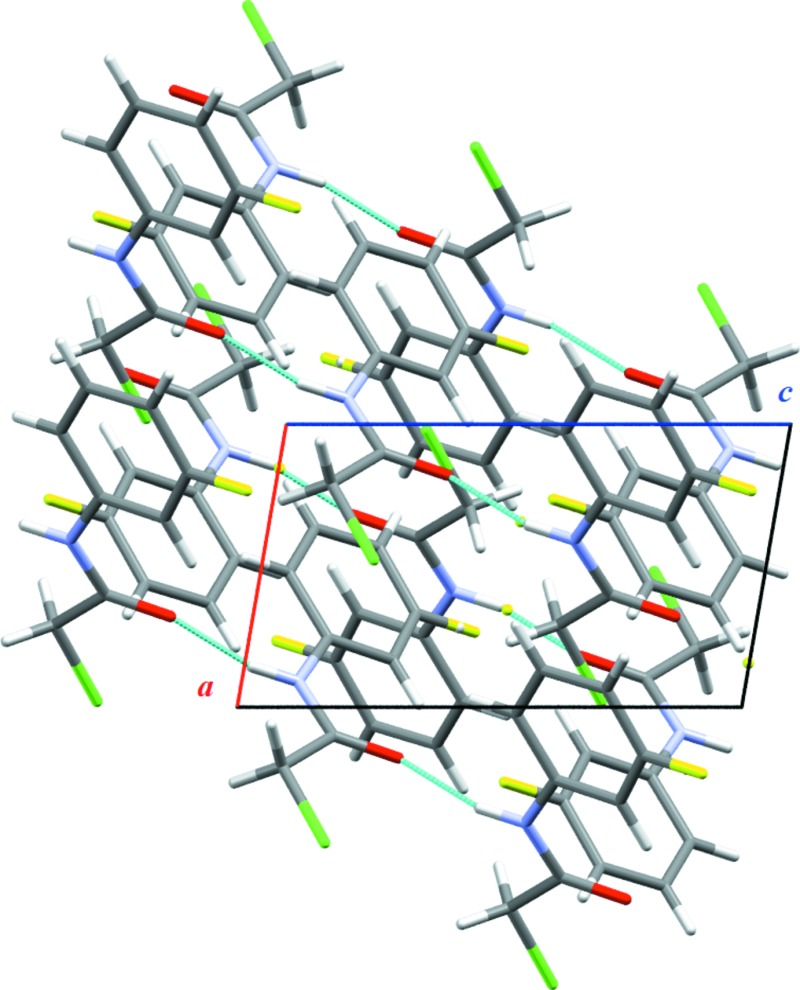
Crystal packing of (I). N—H···O hydrogen bonds are shown as dotted lines.

. DOI: 10.1107/S2056989015007240/hb7400fig2.tif
Crystal packing of (I). N—H⋯O hydrogen bonds are shown as dotted lines.

CCDC reference: 1049536

Additional supporting information:  crystallographic information; 3D view; checkCIF report

## Figures and Tables

**Table 1:** Hydrogen-bond geometry (, )

*D*H*A*	*D*H	H*A*	*D* *A*	*D*H*A*
C2H2O1	0.93	2.33	2.885(3)	118
N1H1O1^i^	0.89(2)	1.99(3)	2.843(2)	160(2)

Symmetry code: (i) 
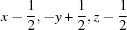
.

## supplementary crystallographic information

### S1. Synthesis and crystallization

The title compound (scheme 1) was synthesized by the reaction of 2-chloro­acetyl chloride with 3-fluoro­aniline at room temperature. The reaction mixture was poured into crushed ice and the resulting solid was washed thoroughly with water, dilute hydro­chloric acid and filtered.

A small portion of the resulting compound was taken in a 10.0 ml beaker and dissolved in a 1:1 ratio of a mixture of EtOH/H_2_O to obtain colourless prisms by a slow evaporation method at ~24°C.

### S2. Refinement

Crystal data, data collection and structure refinement details are summarized in Table 1. The H atoms of the NH groups were located in a difference map and later restrained to N—H = 0.86 (4) Å. The other H atoms were positioned with idealized geometry using a riding model with C—H = 0.93–0.96 Å. All H atoms were refined with isotropic displacement parameters (set to 1.2 times of the *U*eq of the parent atom).

### Crystal data

**Table:**

C_8_H_7_ClFNO	Prism
*M**_r_* = 187.60	*D*_x_ = 1.551 Mg m^−^^3^
Monoclinic, *P*2_1_/*n*	Melting point: 385 K
Hall symbol: -P 2yn	Cu *K*α radiation, λ = 1.54178 Å
*a* = 5.0441 (2) Å	Cell parameters from 1297 reflections
*b* = 18.2374 (7) Å	θ = 5.6–64.3°
*c* = 8.8653 (3) Å	µ = 3.95 mm^−^^1^
β = 99.843 (1)°	*T* = 293 K
*V* = 803.53 (5) Å^3^	Prism, colourless
*Z* = 4	0.31 × 0.24 × 0.19 mm
*F*(000) = 384	

### Data collection

**Table:**

Bruker APEXII diffractometer	1297 reflections with *I* > 2σ(*I*)
Radiation source: fine-focus sealed tube	*R*_int_ = 0.041
Graphite monochromator	θ_max_ = 64.3°, θ_min_ = 5.6°
phi and φ scans	*h* = −5→5
Absorption correction: multi-scan (*SADABS*; Bruker, 2009)	*k* = −21→21
*T*_min_ = 0.368, *T*_max_ = 0.472	*l* = −9→10
6045 measured reflections	1 standard reflections every 1 reflections
1304 independent reflections	intensity decay: 1%

### Refinement

**Table:**

Refinement on *F*^2^	Primary atom site location: structure-invariant direct methods
Least-squares matrix: full	Secondary atom site location: difference Fourier map
*R*[*F*^2^ > 2σ(*F*^2^)] = 0.039	Hydrogen site location: inferred from neighbouring sites
*wR*(*F*^2^) = 0.105	H atoms treated by a mixture of independent and constrained refinement
*S* = 1.15	*w* = 1/[σ^2^(*F*_o_^2^) + (0.0565*P*)^2^ + 0.4965*P*] where *P* = (*F*_o_^2^ + 2*F*_c_^2^)/3
1304 reflections	(Δ/σ)_max_ < 0.001
123 parameters	Δρ_max_ = 0.26 e Å^−^^3^
1 restraint	Δρ_min_ = −0.25 e Å^−^^3^

### Special details

**Table:**

Experimental. Melting point was determined by using open capillary. FT—IR Spectrum was recorded on Jasco FT—IR Spectrometer. ^1^H-NMR and ^13^C-NMR spectra were recorded on Jeol-400 MHz NMR instrument using DMSO-d6 as solvent. Chemical shift values were expressed in δ (p.p.m.) relative to tetramethylsilane (TMS) as an internal reference standard. Mass spectrum of the compound was recorded on Shimadzu LC-2010EV with ESI probe. The analysis of various spectra are as follows.IR wavenumbers (cm^-1^): C=O 1674.9, C—N 1348–1060, N—H 3510–3120, C—N—C 515–409, C—Cl 850–550, C—Cl 650–515. ^1^H-NMR (399.6 MHz, DMSO-d6) δ: 10.49 (s, 1H, NH), 7.57–7,55 (dd, 1H, Ar—H), 7.34–7.27 (m, 2H, Ar—H), 6.88–6.83 (m, 1H, Ar—H), 2.47 (s, 2H, –CH2-). ^13^C-NMR (100 MHz, DMSO-d6) δ: 165.41, 163.76, 140.67, 130.89, 115.54, 110.80, 106.77, 43.92. MS: Predicted Mass: 187.07; Obtained Mass 188.07 (*M*+1).
Geometry. All s.u.'s (except the s.u. in the dihedral angle between two l.s. planes) are estimated using the full covariance matrix. The cell s.u.'s are taken into account individually in the estimation of s.u.'s in distances, angles and torsion angles; correlations between s.u.'s in cell parameters are only used when they are defined by crystal symmetry. An approximate (isotropic) treatment of cell s.u.'s is used for estimating s.u.'s involving l.s. planes.
Refinement. Refinement of *F*^2^ against ALL reflections. The weighted *R*-factor *wR* and goodness of fit *S* are based on *F*^2^, conventional *R*-factors *R* are based on *F*, with *F* set to zero for negative *F*^2^. The threshold expression of *F*^2^ > 2σ(*F*^2^) is used only for calculating *R*-factors(gt) *etc*. and is not relevant to the choice of reflections for refinement. *R*-factors based on *F*^2^ are statistically about twice as large as those based on *F*, and *R*- factors based on ALL data will be even larger.

### Fractional atomic coordinates and isotropic or equivalent isotropic displacement parameters (Å^2^)

**Table:**

	*x*	*y*	*z*	*U*_iso_*/*U*_eq_	Occ. (<1)
H1	−0.643 (5)	0.2133 (14)	0.515 (3)	0.032 (7)*	
Cl1	−0.02090 (10)	0.34713 (3)	0.71994 (6)	0.0279 (2)	
O1	−0.3206 (3)	0.22817 (8)	0.83631 (16)	0.0277 (4)	
N1	−0.5907 (3)	0.19920 (9)	0.61148 (19)	0.0191 (4)	
C1	−0.7435 (4)	0.14033 (11)	0.6567 (2)	0.0193 (4)	
C7	−0.3994 (4)	0.23849 (10)	0.7000 (2)	0.0200 (4)	
C6	−0.9766 (4)	0.12164 (11)	0.5553 (2)	0.0222 (5)	
H6	−1.0266	0.1475	0.4645	0.027*	
C2	−0.6672 (4)	0.10139 (11)	0.7913 (2)	0.0220 (5)	
H2	−0.5110	0.1133	0.8589	0.026*	
C4	−1.0643 (4)	0.02475 (12)	0.7255 (3)	0.0286 (5)	
H4	−1.1717	−0.0135	0.7496	0.034*	
C8	−0.2919 (4)	0.29966 (12)	0.6089 (2)	0.0271 (5)	
H8A	−0.4359	0.3340	0.5733	0.032*	
H8B	−0.2326	0.2787	0.5198	0.032*	
C5	−1.1321 (4)	0.06407 (12)	0.5923 (3)	0.0271 (5)	
H5	−1.2875	0.0516	0.5247	0.033*	0.574 (4)
F1A	−1.3441 (5)	0.04244 (15)	0.5018 (3)	0.0275 (9)	0.426 (4)
C3	−0.8308 (5)	0.04424 (12)	0.8222 (3)	0.0273 (5)	
H3	−0.7808	0.0178	0.9122	0.033*	0.426 (4)
F1	−0.7655 (5)	0.00473 (14)	0.9442 (3)	0.0394 (8)	0.574 (4)

### Atomic displacement parameters (Å^2^)

**Table:**

	*U*^11^	*U*^22^	*U*^33^	*U*^12^	*U*^13^	*U*^23^
Cl1	0.0277 (3)	0.0285 (4)	0.0258 (4)	−0.00754 (18)	−0.0003 (2)	−0.00248 (19)
O1	0.0374 (9)	0.0285 (8)	0.0142 (8)	−0.0067 (6)	−0.0040 (6)	0.0009 (6)
N1	0.0232 (9)	0.0215 (8)	0.0118 (9)	−0.0014 (6)	0.0003 (6)	0.0004 (6)
C1	0.0211 (10)	0.0193 (9)	0.0185 (10)	0.0015 (8)	0.0065 (8)	−0.0045 (8)
C7	0.0231 (10)	0.0214 (10)	0.0148 (10)	0.0020 (8)	0.0017 (8)	−0.0019 (8)
C6	0.0231 (10)	0.0247 (11)	0.0187 (10)	0.0012 (8)	0.0036 (8)	−0.0030 (8)
C2	0.0234 (10)	0.0259 (10)	0.0172 (10)	0.0009 (8)	0.0046 (8)	−0.0022 (8)
C4	0.0306 (12)	0.0244 (11)	0.0348 (13)	−0.0028 (9)	0.0166 (10)	−0.0036 (9)
C8	0.0321 (12)	0.0276 (11)	0.0193 (11)	−0.0079 (9)	−0.0020 (9)	0.0015 (9)
C5	0.0229 (11)	0.0278 (11)	0.0319 (12)	−0.0040 (8)	0.0080 (9)	−0.0111 (9)
F1A	0.0209 (15)	0.0318 (17)	0.0282 (17)	−0.0062 (11)	−0.0001 (11)	−0.0039 (12)
C3	0.0348 (12)	0.0252 (11)	0.0252 (11)	0.0023 (9)	0.0143 (9)	0.0017 (9)
F1	0.0487 (16)	0.0436 (15)	0.0266 (13)	−0.0043 (11)	0.0082 (10)	0.0146 (11)

### Geometric parameters (Å, º)

**Table:**

Cl1—C8	1.768 (2)	C2—H2	0.9300
O1—C7	1.221 (2)	C4—C5	1.374 (3)
N1—C7	1.342 (3)	C4—C3	1.380 (3)
N1—C1	1.419 (3)	C4—H4	0.9300
N1—H1	0.89 (2)	C8—H8A	0.9700
C1—C2	1.386 (3)	C8—H8B	0.9700
C1—C6	1.394 (3)	C5—F1A	1.284 (4)
C7—C8	1.530 (3)	C5—H5	0.9300
C6—C5	1.383 (3)	C3—F1	1.295 (3)
C6—H6	0.9300	C3—H3	0.9300
C2—C3	1.385 (3)		
			
C7—N1—C1	127.59 (17)	C3—C4—H4	121.3
C7—N1—H1	118.2 (17)	C7—C8—Cl1	111.91 (14)
C1—N1—H1	113.9 (17)	C7—C8—H8A	109.2
C2—C1—C6	120.61 (19)	Cl1—C8—H8A	109.2
C2—C1—N1	123.14 (18)	C7—C8—H8B	109.2
C6—C1—N1	116.23 (18)	Cl1—C8—H8B	109.2
O1—C7—N1	125.18 (19)	H8A—C8—H8B	107.9
O1—C7—C8	123.40 (18)	F1A—C5—C4	115.8 (2)
N1—C7—C8	111.42 (16)	F1A—C5—C6	122.1 (2)
C5—C6—C1	118.9 (2)	C4—C5—C6	122.1 (2)
C5—C6—H6	120.6	C4—C5—H5	118.9
C1—C6—H6	120.6	C6—C5—H5	118.9
C3—C2—C1	117.9 (2)	F1—C3—C4	116.4 (2)
C3—C2—H2	121.0	F1—C3—C2	120.6 (2)
C1—C2—H2	121.0	C4—C3—C2	123.1 (2)
C5—C4—C3	117.4 (2)	C4—C3—H3	118.5
C5—C4—H4	121.3	C2—C3—H3	118.5
			
C7—N1—C1—C2	−18.4 (3)	N1—C7—C8—Cl1	−175.19 (14)
C7—N1—C1—C6	163.16 (19)	C3—C4—C5—F1A	177.0 (2)
C1—N1—C7—O1	1.9 (3)	C3—C4—C5—C6	−0.8 (3)
C1—N1—C7—C8	−177.30 (18)	C1—C6—C5—F1A	−177.6 (2)
C2—C1—C6—C5	0.7 (3)	C1—C6—C5—C4	0.0 (3)
N1—C1—C6—C5	179.19 (17)	C5—C4—C3—F1	−177.5 (2)
C6—C1—C2—C3	−0.7 (3)	C5—C4—C3—C2	0.8 (3)
N1—C1—C2—C3	−179.04 (18)	C1—C2—C3—F1	178.1 (2)
O1—C7—C8—Cl1	5.6 (3)	C1—C2—C3—C4	−0.1 (3)

### Hydrogen-bond geometry (Å, º)

**Table:**

*D*—H···*A*	*D*—H	H···*A*	*D*···*A*	*D*—H···*A*
C2—H2···O1	0.93	2.33	2.885 (3)	118
N1—H1···O1^i^	0.89 (2)	1.99 (3)	2.843 (2)	160 (2)

Symmetry code: (i) *x*−1/2, −*y*+1/2, *z*−1/2.
