# Supplementary figures and images for: Crystal structure of 2-chloro-N-(3-fluoro­phen­yl)acetamide
Source: Acta Crystallogr E Crystallogr Commun. 2015 Apr 18;71(Pt 5):o315. doi: 10.1107/S2056989015007240 (PMC4420121; doi:10.1107/S2056989015007240)

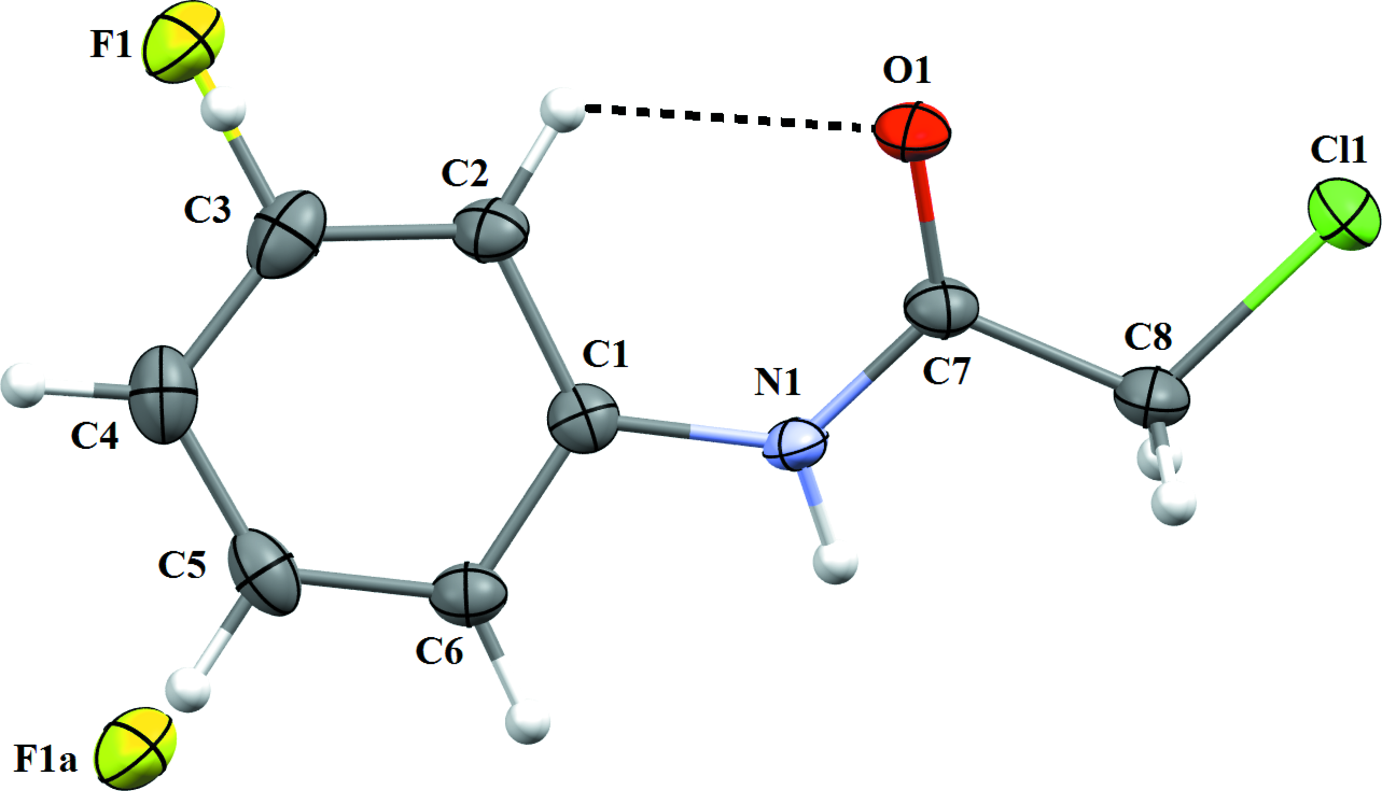

Supplement: Supplementary file 4 [file e-71-0o315-fig1.tif]

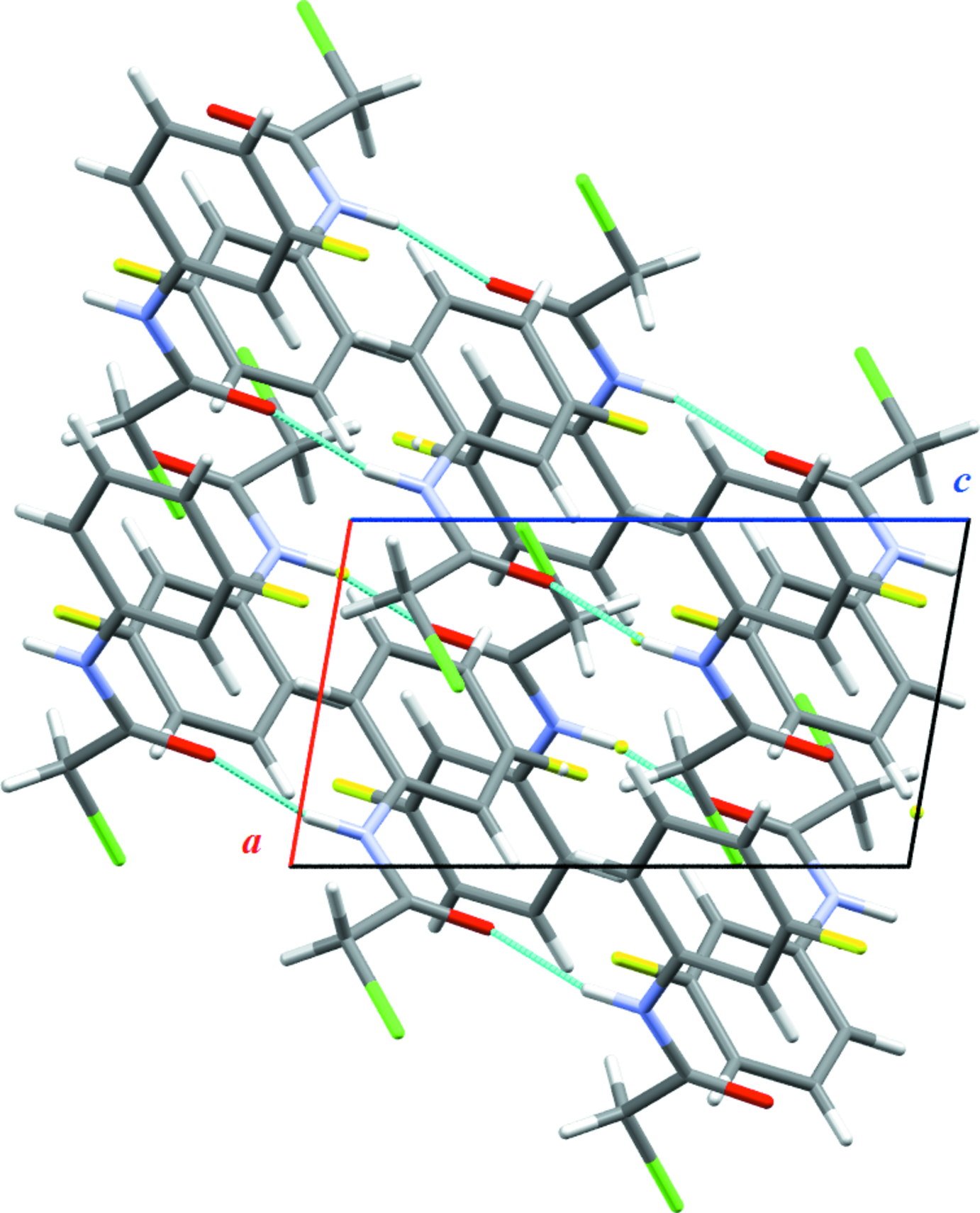

Supplement: Supplementary file 5 [file e-71-0o315-fig2.tif]
